# Supplementary material for: Seismically imaged lithospheric delamination and its controls on the Mesozoic Magmatic Province in South China
Source: Nat Commun. 2023 May 11;14:2718. doi: 10.1038/s41467-023-37855-5 (PMC10175556; doi:10.1038/s41467-023-37855-5)
Supplement: Supplementary file 1 — Supplementary information [file 41467_2023_37855_MOESM1_ESM.pdf]

Supplementary Information for

Seismically imaged lithospheric delamination and its controls on the Mesozoic Magmatic Province in south China

Haijiang Zhang[1,7]*, Qing-Tian Lu[2], Xiao-Lei Wang[3], Shoucheng Han[1], Lijun Liu[4], Lei Gao[1,2], Rui Wang[5], Zeng-Qian Hou[6,2]*

[1]University of Science and Technology of China, School of Earth and Space Sciences, Hefei 230026, China
[2] Chinese MNR Laboratory of Deep Earth Sciences and Technology, Chinese Academy of Geological Sciences, Beijing 100037, China
[3]State Key Laboratory for Mineral Deposits Research, School of Earth Sciences and Engineering, Nanjing University, Nanjing 210023, China
[4]Department of Geology, University of Illinois Urbana-Champaign, United States of America
[5]State Key Laboratory of Geological Processes and Mineral Resources, CUGB, Beijing 100083, China
[6]Institute of Geology, Chinese Academy of Geological Sciences, Beijing 100037, China
[7]Mengcheng National Geophysical Observatory, University of Science and Technology of China, Hefei, Anhui 230026, China

Nature Communications

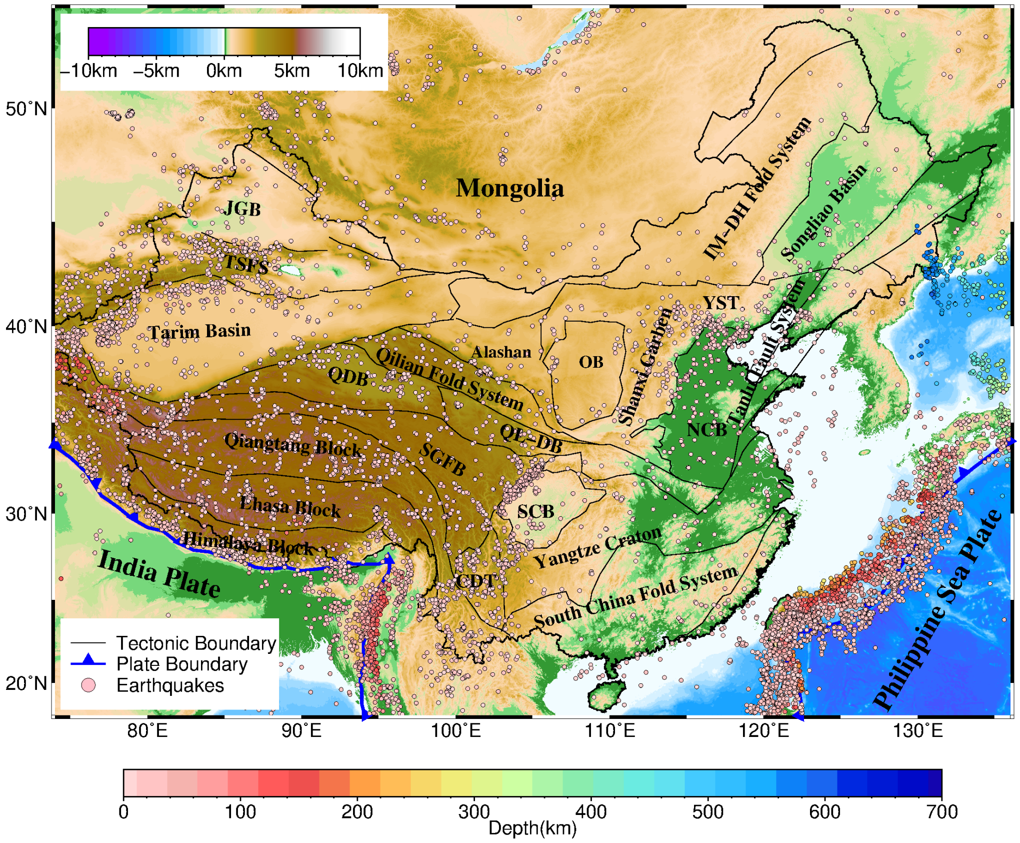

Supplementary Fig. 1. The distribution of earthquakes used in this study with the color legend represents event depths. Plate boundaries are marked by the blue solid lines with triangles and black solid lines depict the boundaries between major tectonic blocks. Abbreviations: *OB:* Ordos basin, *NCB:* North China Block, *QL-DB:* Qinling-Dabie fold system, *SCB:* Sichuan basin, *JGB:* Junggar basin, *TSFS:* Tienshan fold system, *QDB:* Qaidam basin, *SGFB:* Songpan-Ganzi Fold Belt.

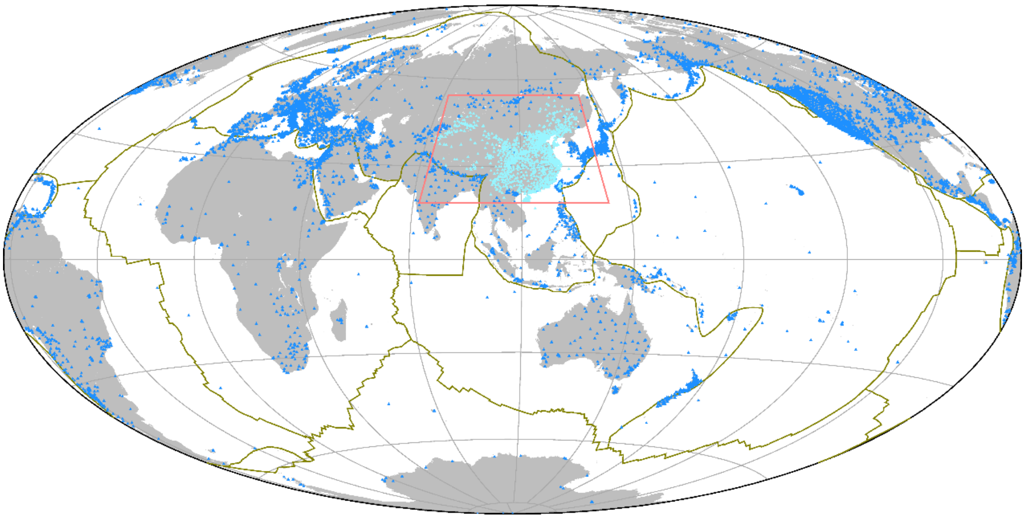

Supplementary Fig. 2. The distribution of seismic stations (blue triangles) used in this study.

The blue triangles are ISC-EHB stations and the light blue triangles are CDSN stations.\

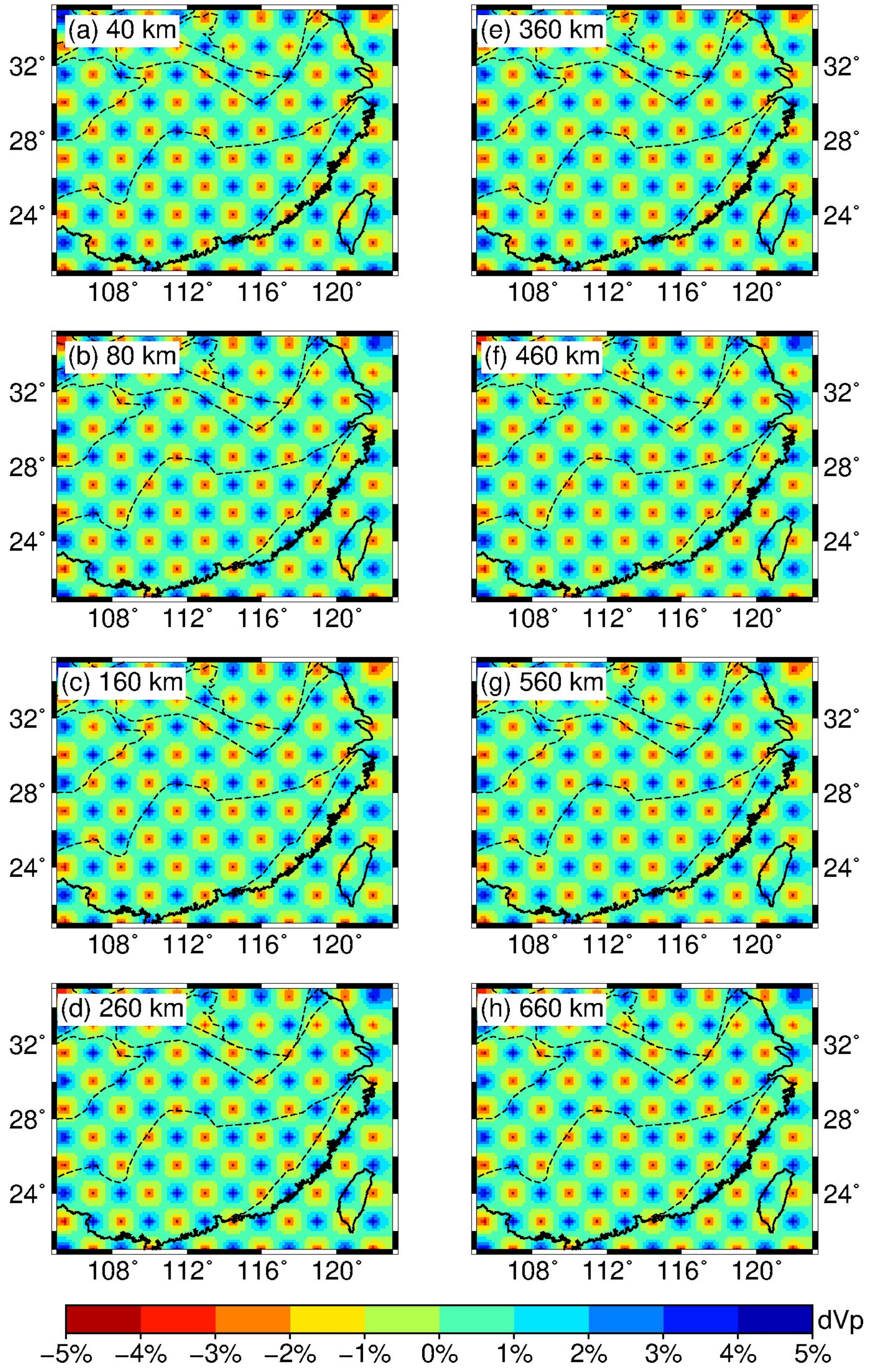

Supplementary Fig. 3. Input checkerboard test patterns at different depths.

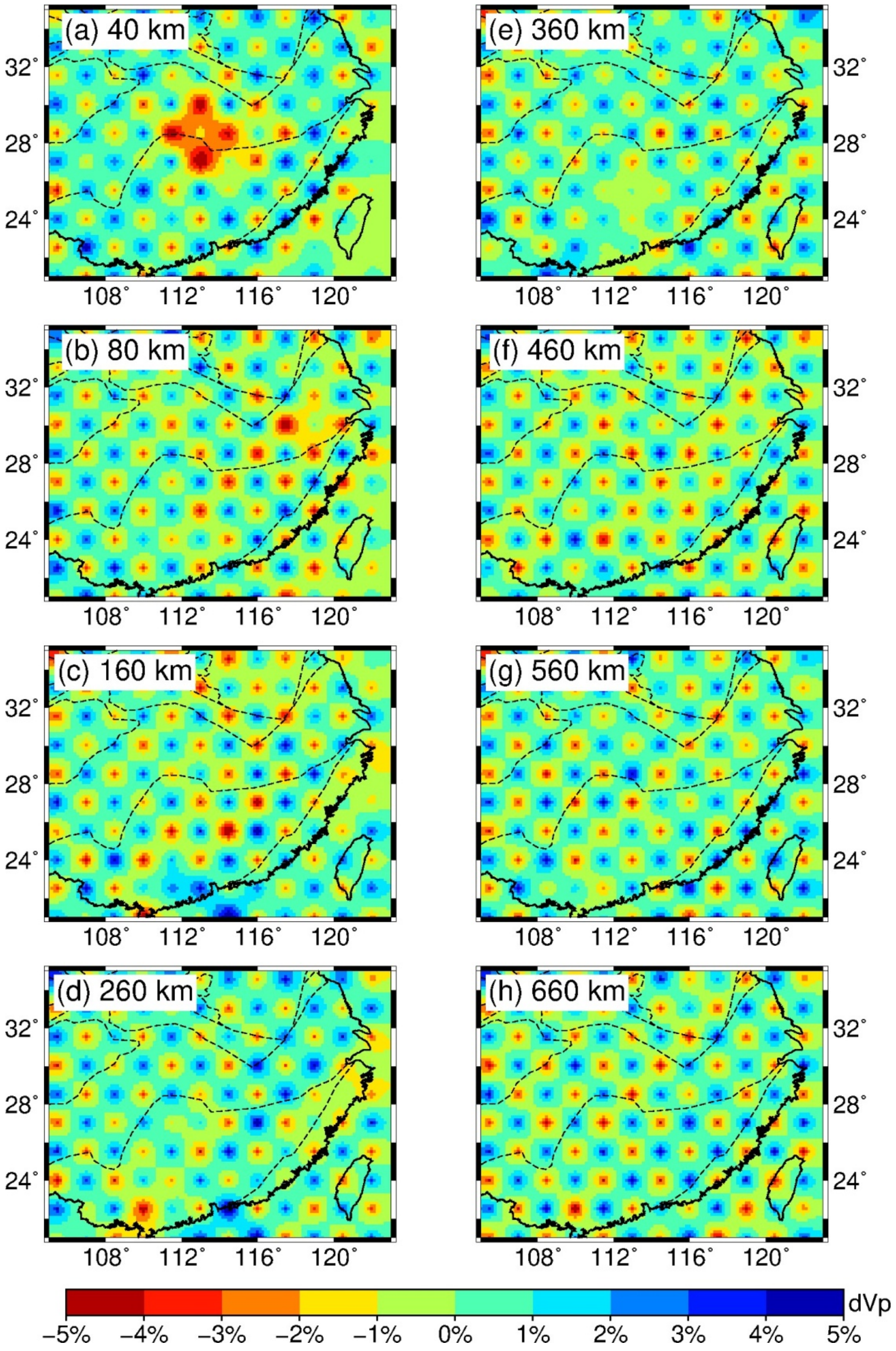

Supplementary Fig. 4. Recovered checkerboard test patterns at different depths.

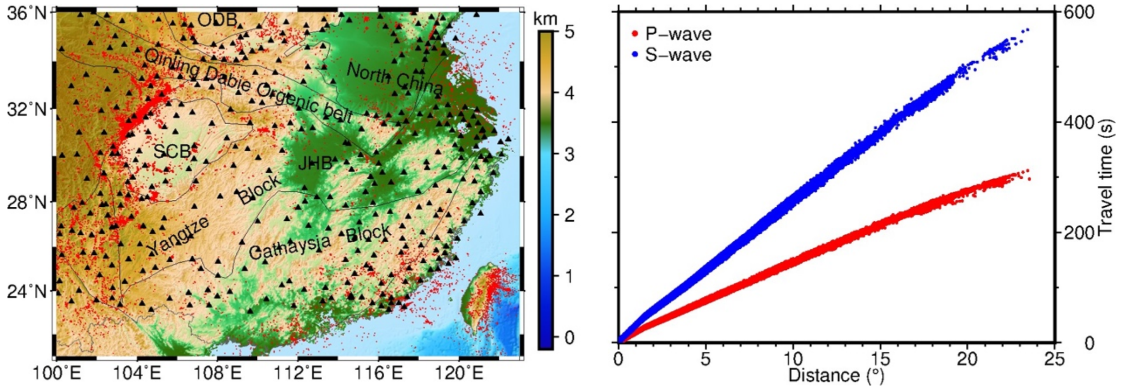

Supplementary Fig. 5. The distribution of seismic stations (black triangles) and earthquakes (red dots) over the topography. The black solid lines indicate the main tectonic boundaries in south China. SCB, Sichuan Basin; ODB, Ordos Block; NCC, North China Craton; JHB: Jianghan Basin.

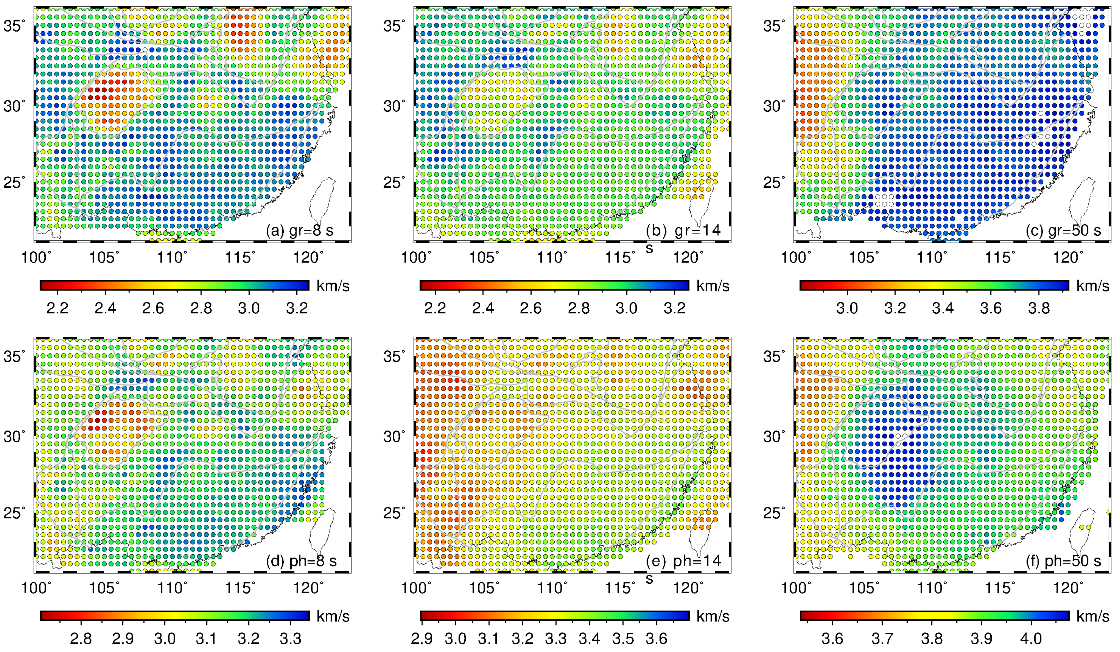

Supplementary Fig. 6. Rayleigh-wave group (a-c) and phase velocity maps (d-f) used in this study with selected periods.

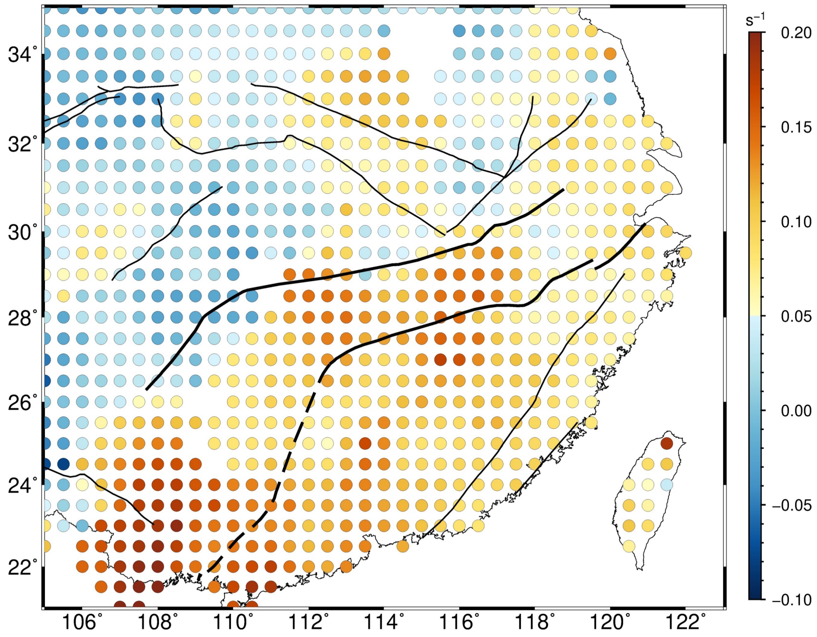

Supplementary Fig. 7. Time slice of smoothed/interpolated receiver function time series at 3 second after the direct P wave. The color indicates the receiver function amplitude. Black dashed lines indicate tectonic boundaries and major faults.

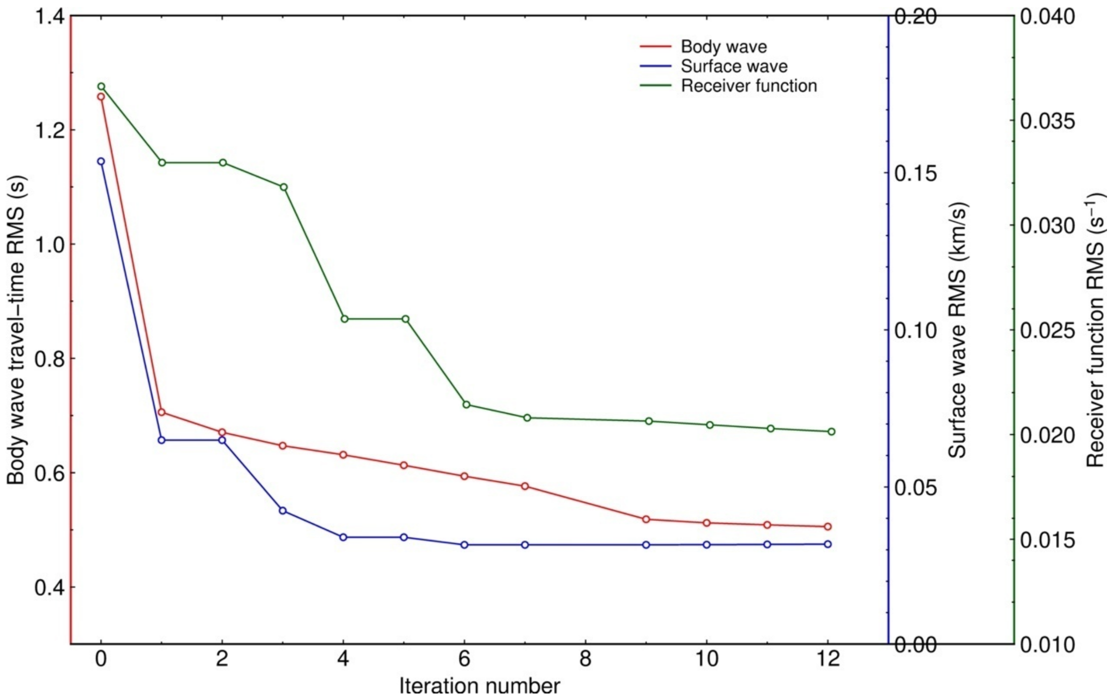

Supplementary Fig. 8. Root mean square (RMS) residuals for body wave (red line), surface wave (blue line) and receiver functions (green line).

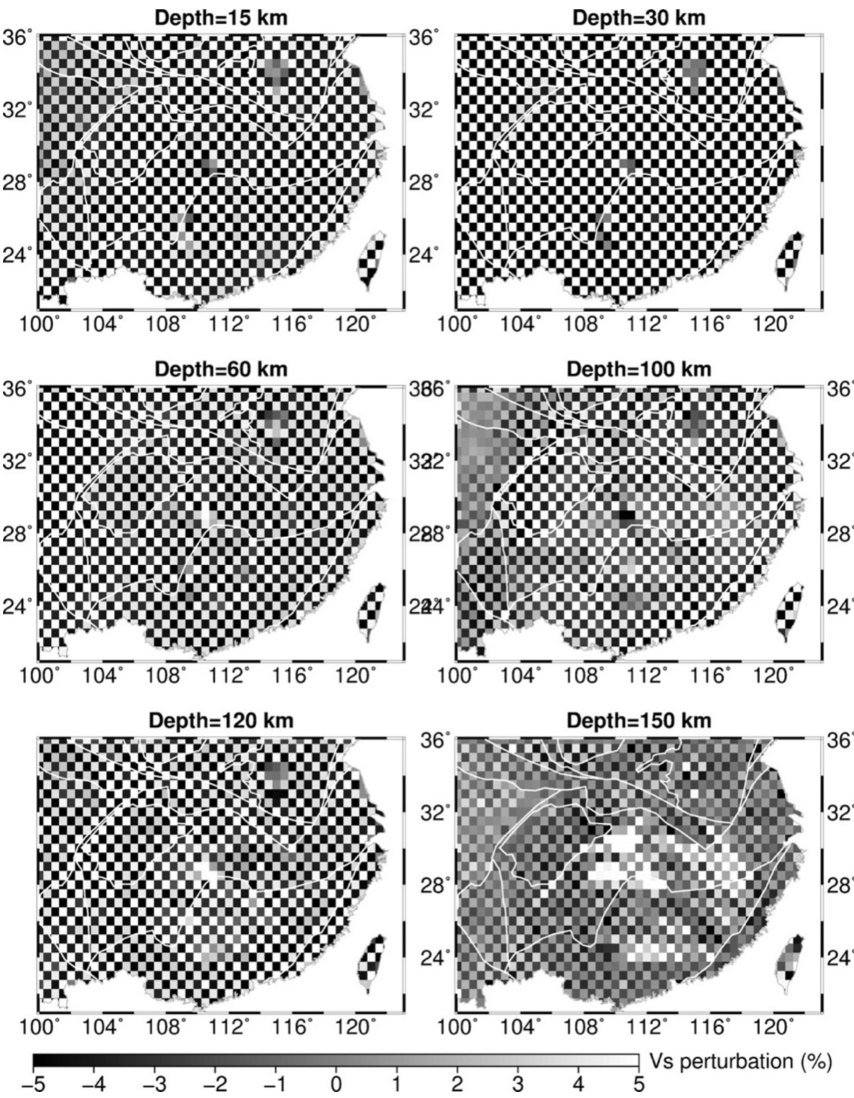

Supplementary Fig. 9. Recovered checkerboard models of S-wave velocities at depths of 15, 30, 60, 100, 120, and 150 km.

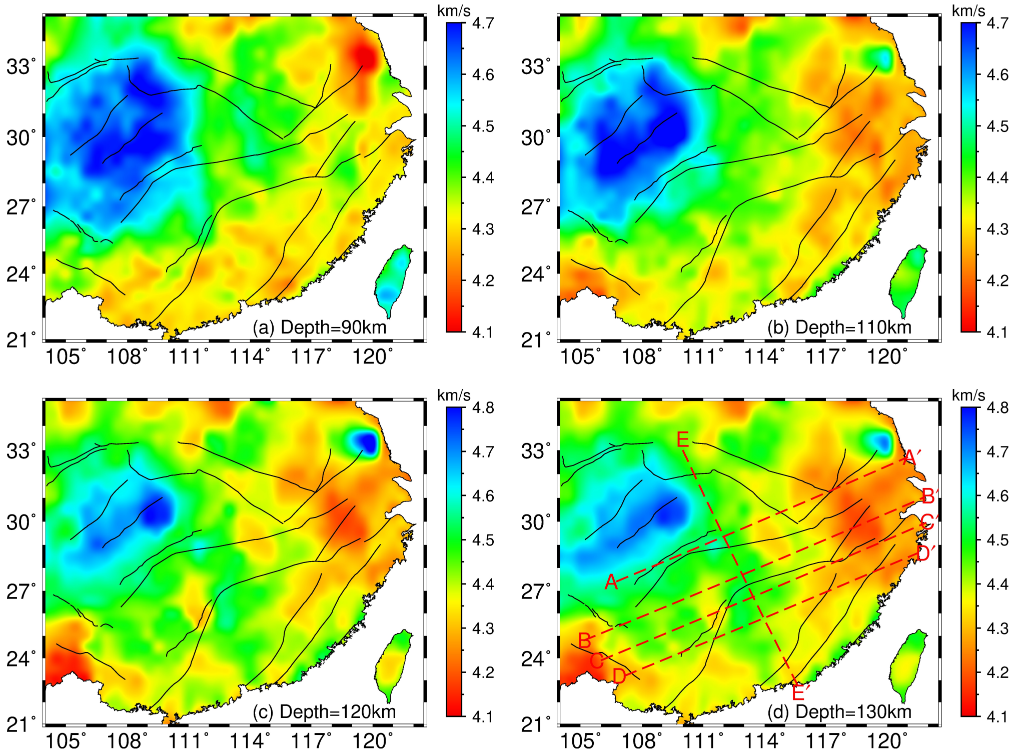

Supplementary Fig. 10. Depth slices of Vs model at depths of 90, 110, 120 and 130 km, respectively.

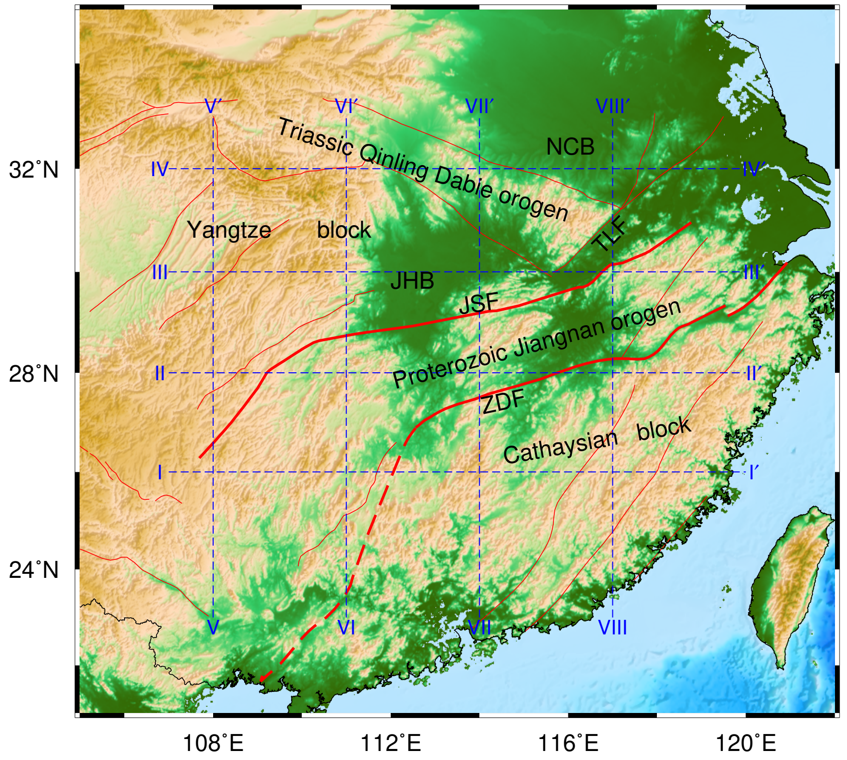

Supplementary Fig. 11. Profile positions for cross sections of the Vs model shown in Supplementary Fig. 12.

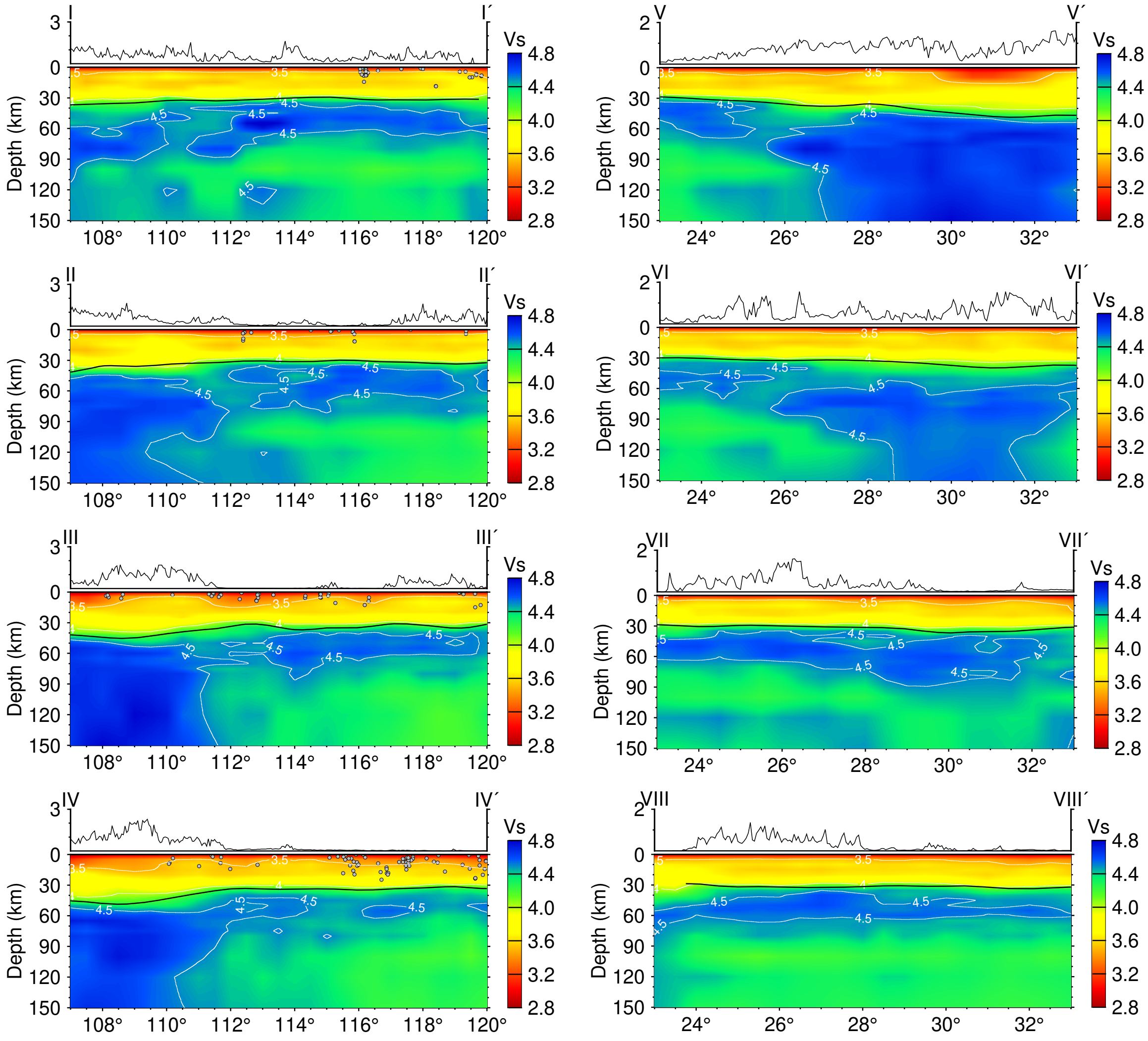

Supplementary Fig. 12. Cross sections of the Vs model along profiles I-XIII shown in Supplementary Fig. 11.

(a)

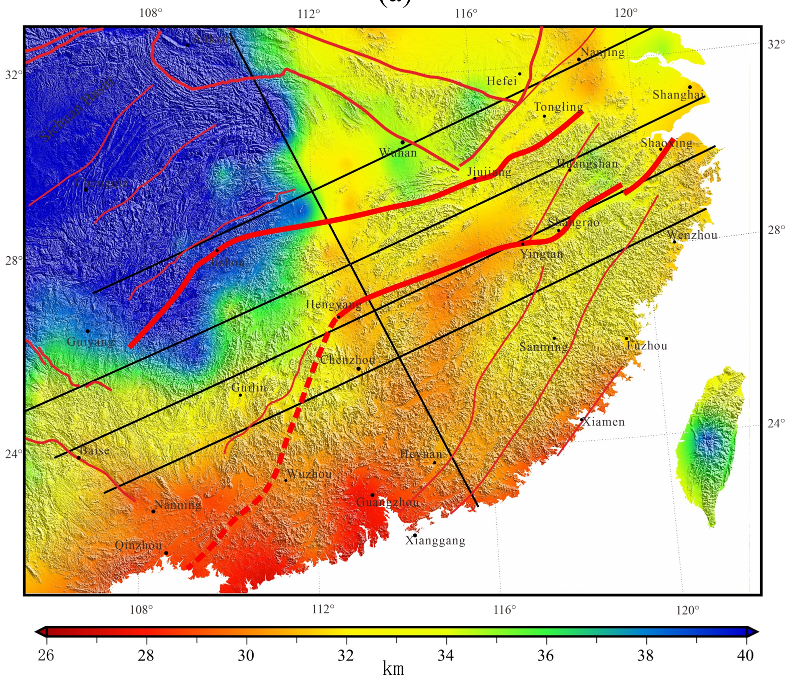

(b)

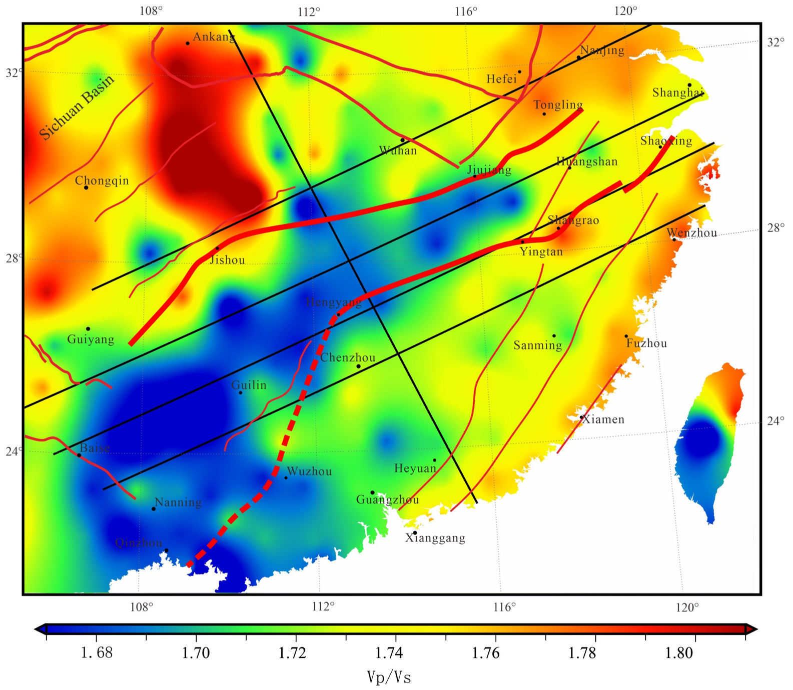

Supplementary Fig. 13. The distribution of Moho depths (a) and average crustal Vp/Vs values (b) in South China that are averaged from results of He et al.[1], He et al.[2], Wei et al.[3], Song et al.[4], Guo et al.[5], Yang & Li[6], and Zhang et al.[7].

References

1. He, C., Dong, S., Santosh, M. & Chen, X. Seismic evidence for a geosuture between the Yangtze and Cathaysia Blocks, South China. *Sci. Rep.* **3,** 1-7 (2013).
2. He, R., Shang, X., Yu, C., Zhang, H. & van der Hilst, R. D. A unified map of Moho depth and Vp/Vs ratio of continental China by receiver function analysis. *Geophys. J. Int.* **199**, 1910-1918 (2014).
3. Wei, Z., Chen, L., Li, Z., Ling, Y. & Li, J. Regional variation in Moho depth and Poisson's ratio beneath eastern China and its tectonic implications. *J. Asian Earth Sci.* **115**, 308-320 (2016).
4. Song, P., Zhang, X., Liu, Y. & Teng, J. Moho imaging based on receiver function analysis with teleseismic wavefield reconstruction: Application to South China. *Tectonophysics* **718**, 118-131 (2017).
5. Guo, L., Gao, R., Shi, L., Huang, Z. & Ma, Y. Crustal thickness and Poisson's ratios of South China revealed from joint inversion of receiver function and gravity data. *Earth Planet. Sci. Lett.* **510**, 142-152 (2019).
6. Yang, X. & Li, Y. Crustal thicknesses and Vp/Vs ratios beneath South China estimated from receiver function analysis and their geological implications. *Chinese J. Geophys.* **64**, 146-156 (2021).
7. Zhang, Y. et al. The crustal thickness and composition in the eastern South China Block constrained by receiver functions: Implications for the geological setting and metallogenesis. *Ore Geol. Rev.* **130,** 103988 (2021).
